# Evolution of Antimicrobial Resistance in Neonatal Sepsis: A Narrative Review

**DOI:** 10.3390/antibiotics15070682

**Published:** 2026-07-11

**Authors:** Nikolaos G. Papanikolaou, Vasileios Giapros, Eleni Papaioannou, Aikaterini I. Nikolaou, Maria Baltogianni, Foteini Balomenou, Maria Kamperi, Niki Dermitzaki

**Affiliations:** 1 School of Medicine, Faculty of Health Sciences, National and Kapodistrian University of Athens, 11527 Athens, Greece; smd2300123@uoa.gr; 2 Neonatal Intensive Care Unit, School of Medicine, University of Ioannina, 45500 Ioannina, Greece; nikaikaterini@gmail.com (A.I.N.); mbalt@doctors.org.uk (M.B.); f.balomenou@uoi.gr (F.B.); mar.kamperi@gmail.com (M.K.); n.dermitzaki@uoi.gr (N.D.); 3 Neonatal Intensive Care Unit, Hippokration General Hospital of Thessaloniki, 54642 Thessaloniki, Greece; epapaioannu@gmail.com

**Keywords:** neonatal sepsis, neonatal intensive care unit, antimicrobial resistance, multidrug-resistant sepsis, antimicrobial stewardship

## Abstract

Antimicrobial resistance is a growing threat in neonatal intensive care units (NICUs) worldwide, challenging the management of neonatal sepsis for decades. The aim of this narrative review is to compare the epidemiology and resistance patterns of neonatal sepsis in NICUs between two periods, 2000–2005 and 2020–2025, and to identify key insights that may inform future practices to limit the emergence and dissemination of antimicrobial resistance in NICUs. During the early 2000s, resistant pathogens, including extended-spectrum beta-lactamases (ESBL)-producing *Enterobacterales*, vancomycin-resistant *Enterococci* (VRE), methicillin-resistant *Staphylococcus aureus* (MRSA), and coagulase-negative *Staphylococci* (CoNS), were increasingly reported in NICUs. The increasing prevalence of antimicrobial resistant strains was associated with the widespread use of broad-spectrum antibiotics, exerting selective pressure that contributed to the emergence of multidrug-resistant pathogens in the 2020s, including carbapenem-resistant *Enterobacterales* (CRE) and multidrug-resistant *Acinetobacter baumannii*, and to the further dissemination of resistant strains in NICUs. The evolution of antimicrobial resistance over the past twenty years highlights that preserving the effectiveness of antibiotics, through rational antibiotic use, is a key strategy to limit the emergence of resistant pathogens. This is of particular importance for the neonatal population due to the limited therapeutic options. Although antimicrobial stewardship programs have been implemented in numerous NICUs with encouraging results, optimization of antibiotic use requires the identification of biomarkers that can promptly and accurately diagnose sepsis and the development of new effective antimicrobial agents against multidrug-resistant pathogens. Future research is expected to improve diagnostic precision, therapeutic options, and stewardship strategies to limit the spread of antimicrobial resistance.

## 1. Introduction

Neonatal sepsis is one of the leading causes of neonatal morbidity and mortality in neonatal intensive care units (NICUs) worldwide [1]. Sepsis disproportionally affects the most preterm and low birth weight neonates, owing to their immature immune system, comorbidities, increased need for invasive procedures, and prolonged hospitalization [2,3]. The prevalence of neonatal sepsis in the United States (US) is reported to be 1 per 1000 live births, with a significantly higher incidence observed among preterm neonates, approximately tenfold higher than that of term neonates [4]. A significant variation in the incidence of neonatal sepsis is observed between different geographic regions. It has been estimated that the global pooled incidence of neonatal sepsis is 22 per 1000 live births, with a mortality rate of 11–19%. The prevalence of sepsis is 40 times higher, and the mortality rates are twice as high in low-and middle-income countries (LMICs) compared to high-income countries (HICs) [5].

Depending on the age of onset, sepsis can be categorized as early-onset sepsis (EOS) or late-onset sepsis (LOS). EOS is defined as sepsis occurring during the first 72 h from birth in hospitalized neonates or the first seven days after birth in nonhospitalized neonates, and LOS, sepsis that occurs thereafter [1]. The acquisition of pathogens in EOS cases is typically vertical, in contrast to the implication of nosocomial or community-acquired pathogens in LOS cases [3].

Prompt diagnosis and antibiotic treatment are critical for improving outcomes of neonatal sepsis. However, the non-specific clinical presentation of neonatal sepsis and the low predictive value of currently available biomarkers result in the overuse of antibiotics in NICUs [1,6]. Overprescription of antibiotics, prolonged antibiotic courses, and broad-spectrum antibiotics are associated with the emergence of resistant pathogens, which represent a growing global threat [6].

The increasing prevalence of multidrug-resistant pathogens in many NICUs worldwide poses a significant challenge to the management of neonatal sepsis [7]. In the 2000s, extended-spectrum beta-lactamase (ESBL)-producing *Enterobacterales*, vancomycin-resistant *Enterococci* (VRE), and methicillin-resistant *Staphylococcus aureus* (MRSA) emerged in NICUs and were implicated in outbreaks [8,9,10]. At present, these pathogens continue to pose significant threats in NICUs, while other multidrug-resistant pathogens, such as carbapenem-resistant *Enterobacterales* (CRE) and multidrug-resistant *Acinetobacter baumannii*, have emerged [7,11].

The management of multidrug-resistant sepsis is challenging due to the limited antimicrobial agents available, representing a major global health concern. Antimicrobial agents authorized for use in neonates are even more restricted [12].

In response to the increasing prevalence of antimicrobial resistance, optimization of antibiotic use in NICUs through antimicrobial stewardship programs is a critical strategy to mitigate the spread of resistance. In parallel, advances in molecular diagnostic techniques can significantly improve pathogen detection and enhance understanding of transmission dynamics, facilitating targeted antibiotic therapy and strengthening of infection prevention efforts [6,13].

Despite advances in infection prevention, diagnostics, and antibiotic development, multidrug-resistant pathogens continue to threaten the effective treatment of neonatal sepsis. It is therefore clinically relevant to consider drivers of the evolution of antimicrobial resistance over time to inform future practices aimed at mitigating the spread of resistant pathogens.

**Figure 1:**
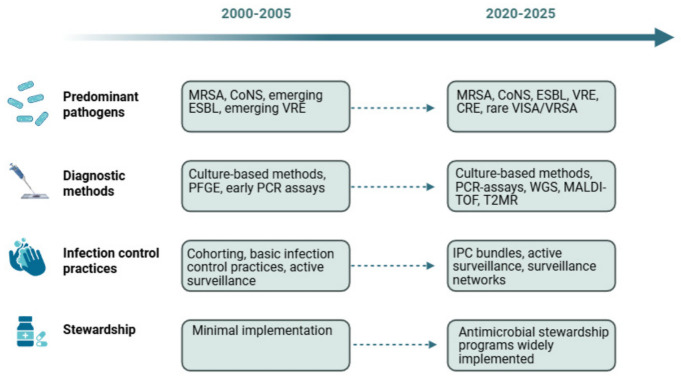
Comparison of resistant pathogens, diagnostic methods, infection control and antimicrobial stewardship between 2000–2005 and 2020–2025. ESBL: expended spectrum beta-lactamases; CRE: carbapenem-resistant *Enterobacterales*; VRE: Vancomycin-resistant *Enterococcus*; MRSA: methicillin-resistant *Staphylococcus aureus*; CoNS: Coagulase-negative *Staphylococcus*; VRSA: vancomycin-resistant *Staphylococcus aureus*; VISA: vancomycin-intermediate *Staphylococcus aureus*; PFGE: pulsed-field electrophoresis; PCR: polymerase-chain reaction; WGS: whole-genome sequencing; MALDI-TOF: matrix-aided laser desorption/ionization time-of-flight mass spectrometry; T2MR: T2 Magnetic Resonance; IPC: infection prevention and control.

The aim of this narrative review is to compare the epidemiology and resistance patterns of neonatal sepsis in NICUs between two representative time periods, 2000–2005 and 2020–2025. These time periods correspond to the early emergence and contemporary era of antimicrobial resistance in NICUs, respectively. This comparison is not intended to provide a temporal analysis of resistance trends, but rather to contrast two clinically relevant time points separated by two decades to highlight major changes in epidemiology, resistance patterns, diagnostic approaches and management strategies (Figure 1). This review provides a clinically focused synthesis of the evolution of antimicrobial resistance in NICUs. It also highlights key lessons from the comparison and discusses future priorities for antimicrobial stewardship, diagnostics, surveillance, and infection prevention strategies to limit the emergence and spread of antimicrobial resistance in NICUs.

## 2. Literature Search Strategy

A structured and comprehensive search was conducted using the online databases PubMed, Scopus, and Google Scholar from February to April 2026 to identify relevant studies published during the periods 2000–2005 and 2020–2025.

The following search terms were used in Pubmed and adapted for the other databases: (“neonatal sepsis” OR “neonatal intensive care unit”) AND (“antibiotic resistance” OR “multidrug-resistant”) AND (“extended-spectrum beta-lactamases” OR “vancomycin-resistant *Enterococcus*” OR “methicillin-resistant *Staphylococcus aureus*” OR “coagulase-negative *Staphylococci*” OR “carbapenem-resistant *Enterobacterales*” OR “*Acinetobacter baumannii*” OR “antimicrobial stewardship” OR “molecular diagnostics”).

Only full-text, peer-reviewed studies written in English were included. Studies focusing on neonates and investigating the epidemiology of neonatal infections, antimicrobial resistance, and the prevention, diagnosis and management of neonatal bacterial sepsis were included. Studies not related to antimicrobial resistance in neonates, involving non-bacterial pathogens and lacking sufficient methodological information were excluded. The reference lists of the retrieved articles were reviewed to assess for the presence of relevant articles that may not have been detected in the initial search.

The article selection, which involved screening titles and abstracts, followed by full-text evaluation and data extraction, was performed independently by two authors. In cases of uncertainty, the decision-making process involved discussion with the co-authors. Due to the narrative nature of the review, a formal tool for risk of bias assessment was not used, which represents a limitation as it prevents a fully systematic evaluation of the included studies. However, potential biases and methodological limitations of the included studies were evaluated.

## 3. Common Pathogens in Neonatal Sepsis

### 3.1. 2000–2005

During the early 2000s, a shift was observed in the causative pathogens implicated in EOS in the neonatal population. Large US cohorts of term and preterm neonates identified Group B *Streptococcus* (GBS) as the leading cause of EOS, followed by *E. coli*. Among preterm neonates, non-GBS infections were more prevalent. However, these studies were conducted prior to the implementation of antenatal GBS screening [14,15]. The 2002 revision of the Centers for Disease Control and Prevention (CDC) guidelines recommended universal GBS screening for all pregnancies at 35–37 weeks of gestation and intrapartum antibiotic prophylaxis for all colonized women [16]. A significant decline in the incidence of GBS EOS was documented in a Spanish NICU following the implementation of intrapartum prophylaxis, from 2.79 to 0.21 cases per 1000 live births [17]. Despite this decline, GBS remained one of the most common causes of EOS, alongside *E. coli*, accounting for 60–80% of cases [18,19]. Less frequently isolated pathogens included *K. pneumoniae*, other *Enterobacterales*, *L. monocytogenes*, and *Enterococcus* species [18].

In very low birth weight (VLBW) neonates, data from the National Institute of Child Health and Human Development (NICHD) Neonatal Research Network indicated a shift toward an increased prevalence of Gram-negative pathogens causing EOS compared with the past decade, during which Gram-positive pathogens predominated [20,21]. *E. coli* was the most prevalent organism isolated (41.2%) [21]. The low rate of GBS infections (11.8%) was likely attributable to the widespread use of intrapartum prophylaxis. The most common gram-positive pathogens were coagulase-negative *Staphylococci* (CoNS), accounting for 14.7% of EOS.

LOS is primarily caused by nosocomial pathogens acquired during NICU hospitalization. *S. aureus*, CoNS, *K. pneumoniae*, and *P. aeruginosa* were the most frequent pathogens. In many institutions, CoNS were the most prevalent causes of LOS, particularly affecting extremely low birth weight (ELBW) neonates [18,22,23]. According to the NICHD, 21% of VLBW neonates had one or more episodes of LOS. Gram-positive organisms were identified in 70% of LOS episodes, with nearly half attributable to CoNS. Of the Gram-negative organisms, *E. coli*, *K. pneumoniae*, *P. aeruginosa*, and *Enterobacter* spp. were identified as the most prevalent pathogens. Fungal infections, predominantly due to *Candida* species, accounted for approximately 12% of LOS episodes [24].

### 3.2. 2020–2025

In HICs, the pathogens most commonly implicated in neonatal sepsis have remained largely unchanged over the past few decades. Despite the considerable reduction in the prevalence of GBS sepsis following the implementation of routine intrapartum screening and antibiotic prophylaxis, GBS remains the leading cause of EOS, followed by *E. coli*. It is estimated that approximately two-thirds of EOS cases are attributable to these two pathogens in HICs [1,3,7,25].

As in the early 2000s, in HICs, *E. coli* remains the leading cause of EOS in the preterm population, accounting for approximately 50% of cases, whereas GBS accounts for about 20% of EOS episodes [4,25]. In a large US prospective surveillance study, Stoll et al. reported an increased prevalence of *E. coli* infections in VLBW neonates compared with the previous decade (8.68 vs. 5.07 per 1000 live births; *p* = 0.008) [26]. The remaining EOS cases are caused by other Gram-positive pathogens, including *S. aureus*, *Enterococcus* spp., and *L. monocytogenes*, as well as Gram-negative pathogens, such as *Klebsiella*, *Enterobacter*, and *Haemophilus* species [4,25].

Although the burden of LOS, particularly in preterm neonates, remains substantial, a decline in the incidence has been observed compared to previous decades in HICs [27,28]. Gram-positive pathogens remain the most prevalent, predominantly CoNS, and less frequently, *S. aureus*, *Enterococcus* spp., and GBS [1,25,29]. Gram-negative pathogens account for 20–30% of LOS, with *E. coli* and *K. pneumoniae* being the most common, and, to a lesser extent, *S. marcescens*, *Enterobacter* spp., and *P. aeruginosa* [3,25,29]. Approximately 5% of LOS episodes in extremely preterm neonates involve *Candida* spp. [28]. However, significant variation exists in the pathogens most commonly implicated in LOS across different geographical regions and even within the same region. Gram-positive pathogens are more frequently implicated in LOS in HICs, while Gram-negative pathogens predominate in LMICs [29,30].

## 4. Antimicrobial Resistance

### 4.1. 2000–2005

During the early 2000s, antimicrobial resistance became increasingly prevalent in NICUs, particularly among Gram-negative organisms. Resistance to third-generation cephalosporins and broad-spectrum penicillins increased during this period. Moreover, resistance to aminoglycosides was documented, while resistance to carbapenems and quinolones remained sporadic [31].

The evolution of antimicrobial resistance is influenced by antibiotic prescribing practices [31,32]. The most widely used empirical antibiotic regimen was ampicillin plus an aminoglycoside or third-generation cephalosporin [32]. Ampicillin and third-generation cephalosporins are associated with the selection of Gram-negative pathogens that produce ESBLs, rendering them resistant to a variety of antibiotics, including beta-lactams [33].

An increasing prevalence of pathogens resistant to the widely used EOS regimen, ampicillin, and gentamicin, was documented in studies from the US and Europe. High rates of ampicillin resistance among *E. coli* isolates were already evident in the early 2000s, likely attributable to the high rates of ampicillin intrapartum prophylaxis and the widespread use of ampicillin as an empiric regimen in neonates with suspected sepsis [14,17,21,34,35]. The 2005 NICHD report on EOS in VLBW neonates documented 77% ampicillin resistance among *E. coli* isolates [21]. Furthermore, Alarcon et al. reported an increased prevalence of ampicillin-resistant *E. coli* isolates causing EOS in preterm neonates over a 10-year period (25% vs. 91%); however, no association with the administration of intrapartum prophylaxis could be established. Interestingly, during the same period, the authors reported no increase in the prevalence of ampicillin-resistant *E. coli* isolates causing EOS in term neonates [17]. This is in accordance with the results of other studies [14].

Resistance to aminoglycosides was reported less frequently; however, concerns regarding the efficacy of standard empirical regimens were raised. Gentamicin resistance among *E. coli* isolates was documented in approximately 8–12% of cases [17,21].

The selective pressure exerted by commonly used antibiotics in NICUs became an increasing concern during that period. Moreover, the emergence of specific resistance mechanisms, including ESBLs in Gram-negative pathogens and vancomycin resistance among enterococci, as well as MRSA, posed significant challenges for clinical management in NICUs.

### 4.2. 2020–2025

The recommended empirical regimen for EOS, ampicillin and gentamicin, has remained unchanged over the last decades. However, high levels of ampicillin resistance, documented since the 2000s among *E. coli* isolates, persist, with increasing reports of gentamicin resistance [1]. Flannery et al. reported a 76.6% resistance rate to ampicillin among Gram-negative pathogens implicated in EOS, 8.5% to gentamicin, and 7.3% to both agents in US NICUs. All Gram-positive pathogens were susceptible to ampicillin and/or gentamicin [36]. Another large multicenter US study documented 70% resistance to ampicillin and 16.8% to aminoglycosides in *E. coli* isolates from neonates admitted to NICUs. Among *E. coli* isolates associated with EOS, resistance to both ampicillin and gentamicin was observed in 10% of cases [37]. Stoll et al. reported that ampicillin-resistant *E. coli* was more frequent among preterm compared to term neonates (83.1% vs. 37.5%, *p*  <  0.001) [26]. Recent large observational studies from LMICs have demonstrated that more than 70% of gram-negative pathogens implicated in neonatal sepsis are resistant to at least one beta-lactam and one aminoglycoside [38,39]. In light of increasing antimicrobial resistance, pediatric infectious disease specialists have suggested that while ampicillin and gentamicin remain reasonable empirical regimens for term and most preterm neonates, we are approaching a point at which we should consider alternative empirical regimens at the local or national level [37,40]. However, broader-spectrum antibiotics may be appropriate for critically ill, particularly VLBW neonates, at higher risk for EOS [26,37,40].

A higher level of heterogeneity is reported among antibiotics used to treat LOS [41]. Vancomycin in combination with an aminoglycoside or a third-generation cephalosporin is the most commonly employed antibiotic regimen, while beta-lactams/beta-lactamase inhibitors, carbapenems, and linezolid are also used [29,41]. In multiple regions worldwide, the widespread use of vancomycin, third- and fourth-generation cephalosporins, and carbapenems has led to the emergence of multidrug-resistant pathogens [30].

The prevalence of antimicrobial resistant pathogens has increased globally compared with previous decades. ESBL-producing *Enterobacterales* have been recognized as a serious threat, and CRE and multidrug-resistant *P. aeruginosa* as urgent threats according to the CDC 2019 report [42]. Compared with the early 2000s, when ESBL-producing *Enterobacterales* and VRE were emerging, the current era is characterized by the increasing dissemination of multidrug-resistant pathogens, including CRE, ESBL-producing pathogens, VRE, and MRSA, posing major challenges for many NICUs due to their outbreak potential and limited therapeutic options.

## 5. Multidrug-Resistant Pathogens

### 5.1. ESBL-Producing Enterobacterales

#### 5.1.1. 2000–2005

Until the 1980s, resistance to beta-lactam antibiotics was observed only in pathogens harboring chromosomal beta-lactamase genes, a form of resistance that is not transmissible. In 1983, Knothe et al. described strains of nosocomial *Enterobacterales* demonstrating transmissible, plasmid-mediated resistance to cephalosporins [43]. Over the following years, ESBLs emerged among *Enterobacterales*, particularly *Klebsiella* spp. and *E. coli*, as a global concern [44]. Outbreaks in intensive care units caused by ESBL-producing pathogens were increasingly reported [45].

During the early 2000s, the prevalence of ESBL-producing bacteria varied significantly across geographic regions and even among centers within the same region [46]. In the US, according to the CDC National Nosocomial Infections Surveillance (NNIS), the national average for ESBL-producing *Enterobacterales* was low, at approximately 3%, although rates varied widely between centers (0–25%) [46]. In European countries, significant heterogeneity was also observed. In the Netherlands, very low prevalence (<1%) was reported, whereas substantially higher rates were documented in other countries, such as France, where up to 40% of *K. pneumoniae* isolates were ceftazidime-resistant [46]. Similarly, in Asia, prevalence ranged from negligible levels in Japan to approximately 12% in China [47,48]. Of particular concern was the rapid increase in ESBL infections over a short period. For instance, data from Taiwan demonstrated a substantial increase in the rate of ESBL-producing *Enterobacterales* infections in pediatric intensive care units (PICUs) and NICUs, from 11% of nosocomial infections in 1999 to 44% in 2001 [49].

Although data specific to NICUs during this period were relatively limited, studies from various geographic regions demonstrated a substantial burden of ESBL-producing pathogens in both colonization and infection. In a US NICU, 41% of neonates who developed antimicrobial-non-susceptible *Enterobacterales* sepsis were infected with ESBL-producing strains. Of particular note, 91% of *Klebsiella* spp. and 73% of *E. aerogenes* were ESBL producers [50]. Pessoa-Silva et al. observed that 53.8% of 383 neonates admitted to a level II/III NICU in Brazil were colonized with ESBL-producing *K. pneumoniae* [51]. Similarly, an increased prevalence was reported in Asian NICUs. A Chinese study involving a two-year observation period in a NICU reported that 56.4% of infections caused by *K. pneumoniae* and *E. coli* were due to ESBL-producing strains [52]. Colonization rates among neonates ranged from approximately 20% to over 50% with *K. pneumoniae* predominating. In a Malaysian NICU, 21.7% of admitted neonates were colonized with ESBL-producing *K. pneumoniae* at a median age of nine days [53]. A study from Turkey examined the resistance of the fecal flora of 118 neonates, including those admitted to the NICU and the neonatal ward, as well as healthy term neonates, and reported a 33.7% ESBL colonization rate. Of the *K. pneumoniae* and *E. coli* isolates evaluated, 44.8% and 45.1%, respectively, were identified as ESBL-producing. Interestingly, no difference in the ESBL colonization rate was observed between hospitalized and healthy neonates [54].

In 2005, more than 200 ESBLs had been identified [55]. Whereas TEM- and SHV-producing ESBLs were predominant in previous decades, CTX-M-producing ESBLs emerged as the most frequent genotype in the early 2000s across various geographic regions [56,57]. However, molecular data on ESBL genotypes in NICUs were scarce. Consistent with the broader epidemiology of ESBLs during that period, Wu et al. analyzed 88 ESBL-producing isolates using restriction site polymerase chain reaction (PCR) and reported CTX-M-3 as the predominant genotype among *E. coli* and *K. pneumoniae*. In all *E. cloacae* isolated, SHV-12 was identified [45].

While some consistency in the antimicrobial susceptibility of ESBL-producing pathogens across institutions was noted, variability was also observed. Carbapenems exhibited high levels of activity against ESBL-producing *Enterobacterales* and were considered the most effective therapeutic option [49,52]. Although susceptibility to quinolones was demonstrated in most instances, emerging resistance was reported in several studies [10,49,52]. Moreover, ESBL isolates that exhibited increased resistance to beta-lactam/beta-lactamase inhibitor combinations, including amoxicillin/clavulanic acid, cefoperazone/sulbactam, and ticarcillin/clavulanic acid, were reported and demonstrated to be associated with high-level ESBL production [52].

#### 5.1.2. 2020–2025

Between 2020 and 2025, studies from various geographic regions continued to identify ESBL-producing pathogens as a significant threat. The prevalence of ESBL colonization and infection varies significantly across regions, with higher rates in LMICs [11]. Reports of NICU outbreaks are increasing across multiple geographic regions [58,59,60,61,62]. The endemic presence of ESBL in numerous NICUs has become a notable concern, particularly in LMICs [11].

Recent surveillance studies have highlighted the substantial burden of ESBL colonization in neonatal populations, particularly in LMICs. In a Moroccan NICU, approximately 55% of admitted neonates were colonized by ESBL *K. pneumoniae* [63]. Similarly, a recent Iranian systematic review and meta-analysis reported a 57% third-generation cephalosporin resistance among Gram-negative isolates implicated in neonatal sepsis [64]. A recent meta-analysis of studies conducted in LMICs reported a pooled prevalence of third-generation cephalosporin-resistant *Enterobacterales* colonization of 30.2%, rising to 48.2% when including only hospitalized neonates [65]. In contrast, substantially lower colonization rates have been reported in several HICs. In an Irish NICU, 9% of very preterm neonates were found to be colonized with ESBL-producing isolates, whereas the prevalence of colonization remained low in countries such as the US and Japan [7,66,67].

The CTX-M genotype, which emerged in the early 2000s, has become the predominant type in recent years, particularly CTX-M-15, followed by SHV and TEM genotypes [7,25,58,60,66].

ESBL-producing pathogens are typically resistant to a broad range of antibiotics, including penicillins and cephalosporins. Although ESBLs typically do not inactivate non-beta-lactam antibiotics, pathogens often harbor additional genes or mutations that confer resistance to various agents, including fluoroquinolones and aminoglycosides. Carbapenems are generally effective against ESBL-producing *Enterobacterales* and are the treatment of choice for invasive infections [7,68,69]. The widespread use of carbapenems, driven by the rising prevalence of ESBL-producing pathogens, has raised concerns about selection pressure and the subsequent emergence of carbapenem-resistant *Enterobacterales* in NICUs. Newer beta-lactam/beta-lactamases inhibitor combinations, such as ceftazidime/avibactam and ceftolozane/tazobactam are approved for neonatal use and are effective against ESBLs, however their use should be reserved to preserve their effectiveness [69,70].

### 5.2. CRE

#### 5.2.1. 2000–2005

During the early 2000s, *Enterobacterales* resistance to carbapenems was rare and only sporadically reported [71,72]. However, the increasing prevalence of ESBL-producing *Enterobacterales* was associated with increased carbapenem use during this period. The resulting selective pressure raised concerns about the emergence of carbapenem-resistant strains in NICUs [52].

#### 5.2.2. 2020–2025

The steadily increasing prevalence of ESBL-producing and other multidrug-resistant pathogens has led to an increased use of carbapenems, particularly meropenem and imipenem, in NICUs, thereby contributing to the emergence of carbapenem-resistant pathogens [11]. Carbapenem resistance may be mediated by the production of carbapenemases, such as *Klebsiella pneumoniae* carbapenemase (KPC), New Delhi metallo-beta-lactamase (NDM), Verona integron-encoded metallo-beta-lactamase (VIM), and oxacillinase OXA-48-like enzymes, or production of ESBL or AmpC beta-lactamase combined with structural mutation leading to impaired membrane permeability [7,69,73]. Although carbapenem resistance has been reported in several *Enterobacterales* species, *K. pneumoniae* remains the most frequently isolated pathogen [7,65].

Recent studies have demonstrated an increasing prevalence of CRE colonization and infections in NICUs, particularly in LMICs (Table 1). A Chinese multicenter study reported that 13% of sepsis cases caused by *Enterobacterales* in very preterm neonates were attributable to carbapenem-resistant strains [74]. According to a recent systematic review, the pooled global prevalence of carbapenem-resistant *K. pneumoniae* sepsis in neonates admitted to the NICU is 0.3%. Notably, the analysis included 23 studies, all of which were conducted in LMICs [75].

High colonization rates have been reported in several regions, especially in LMICs. Prematurity, invasive procedures, prolonged antibiotic courses, and prolonged hospitalization have been identified as risk factors for colonization. Moreover, a correlation has been demonstrated between prior carbapenem use and colonization with carbapenemase-resistant strains [76,77]. Studies conducted in African countries, Morocco and Tanzania, have reported a colonization rate of 30% [78]. High colonization rates of 25% have also been documented in a Serbian NICU [76]. In India, colonization rates have been reported to be 5–9% among hospitalized neonates [79,80]. A recent meta-analysis of 26 studies conducted in LMICs demonstrated a pooled prevalence of 6.3% for CRE colonization in hospitalized neonates [65]. Data on CRE colonization in HICs are limited. However, available evidence suggests a significantly lower prevalence than in LMICs [81,82].

Molecular studies have demonstrated the predominance of carbapenemase-producing strains in isolates from NICUs [83,84]. Geographic variations exist in the prevalence of different carbapenemase genes [75,83]. In Saudi Arabia, 71.2% of CRE *K. pneumoniae* isolates encoded OXA-48 type carbapenemases, followed by NDM-1 (20.5%) [85]. The predominance of the NDM gene has been demonstrated in studies conducted in China [77,84]. KPC and VIM carbapenemases were most common in a Portuguese NICU, whereas NDM was most prevalent in Italy [82,86]. A global systematic review reported that approximately 65% of carbapenem-resistant *K. pneumoniae* isolates implicated in neonatal sepsis encode NDM [75]. Notably, the presence of co-harbored carbapenemase genes has been frequently described [83,87].

The global emergence of carbapenem-resistant strains is a significant concern, primarily due to the limited therapeutic options. According to the World Health Organization (WHO), carbapenem-resistant pathogens are classified as a high-priority for antimicrobial research [73]. In neonates, therapeutic options are considerably more limited [88]. Older drugs such as polymyxins and newer drugs such as ceftazidime/avibactam have been used in the treatment of CRE [89].

**Table 1:** Epidemiological and molecular characteristics of carbapenem-resistant Enterobacterales in neonatal intensive care units.

Author	Country	Population	CRE %	CRE Species	CRE Genotyping
Labi, 2020 [90]	Ghana	228	7.9	*Klebsiella pneumoniae* 18/18	OXA-181: 100%
Yin, 2021 [91]	China	1230	11.7	*Klebsiella pneumoniae* 110/144 *Escherichia coli* 29/144 *Enterobacter cloacae* 5/144	NDM-1: 82.7% OXA-23: 46.5% NDM-5: 15.5% KPC: 1.7%
Almeida, 2021 [82]	Portugal	173	5.8	*Klebsiella pneumoniae* 7/10 *Enterobacter cloacae* 3/10	KPC: 40% VIM: 40% OXA-48: 20%
Wang, 2022 [92]	China	1650	14.2	*Klebsiella pneumoniae* *Escherichia coli*	NDM: 94.4%
Agosta, 2023 [86]	Italy	230	7.4	*Escherichia coli* 12/20 *Klebsiella pneumoniae* 8/20	NDM: 100%
Demir, 2023 [83]	Turkey	206	24.3	*Klebsiella pneumoniae* 44/50 *Klebsiella oxytoca* 6/50	NDM: 42% OXA-48: 16% VIM: 2% NDM + OXA-48: 36% No genes detected: 4%
Edwards, 2023 [93]	Nigeria, Kenya	42	62.4	ND	NDM: 95.2% OXA-48: 4.8%
Mussa, 2023 [87]	Morocco	339	22.1	*Klebsiella pneumoniae*	OXA-48: 100% NDM: 30.8% VIM: 9.9% KPC: 2.2%
Mijac, 2023 [76]	Serbia	350	25.1	*Klebsiella pneumoniae* 87/88 *Escherichia coli* 1/88	KCP: 51.1% OXA-48: 47.7% NDM: 1.2%
Guan, 2025 [94]	China	1108	7.3	*Klebsiella pneumoniae*	KCP-2: 45.7% NDM-1: 40.7% NDM-5: 13.6%
Naburi, 2025 [78]	Tanzania	51	29.4	*Klebsiella pneumoniae* 10/15 *Escherichia coli* 4/15 *Klebsiella oxytoca* 1/15	NDM-5: 100% OXA-181: 53%
Dihn, 2026 [95]	Vietnam	36	38.9	*Klebsiella pneumoniae* 6/17 *Enterobacter cloacae* 6/17 *Escherichia coli* 4/17	

KPC: *Klebsiella pneumoniae* carbapenemase; NDM: New Delhi metallo-beta-lactamase; OXA: oxacillinase; VIM: Verona integron-encoded metallo-beta-lactamase.

### 5.3. VRE

#### 5.3.1. 2000–2005

In the early 2000s, the increasing prevalence of VRE emerged as another important threat in NICUs. The first isolated strains of VRE were reported in Europe in 1986 [96]. Over the following years, the prevalence of VRE increased worldwide, establishing VRE as important nosocomial pathogens, particularly in intensive care units [96]. According to the NNIS report, in 2004, 28.5% of enterococcal infections in intensive care units were attributable to VRE strains [97].

During this period, outbreaks of VRE in intensive care units were increasingly reported in the US, Europe and Asia [98]. Data on the burden of VRE in NICUs remained limited, with a focus primarily on colonization rather than infection. Several studies highlighted the potential for VRE outbreaks in NICUs in both LMICs and HICs (Table 2). Khan et al. reported the first 10 VRE isolates of the same clone in a tertiary hospital in Pakistan. Four of the isolates were sourced from the NICU, while six were obtained from the intensive care unit. Although the two units were located in different facilities, they shared a number of personnel, highlighting the healthcare-associated spread of the pathogen [99]. Moreover, in a Turkish study, Yüce et al. collected rectal swabs from neonates admitted to the NICU and from healthy neonates in the obstetric ward to evaluate the incidence and risk factors for VRE colonization. None of the healthy neonates demonstrated VRE colonization, whereas 8/110 hospitalized neonates did. Prolonged hospitalization and prior use of broad-spectrum antibiotics were identified as risk factors for colonization [98]. Outbreak reports highlighted the increasing burden of VRE infections in NICUs in several HICs, such as the US and European countries. Borgmann et al. described two outbreaks caused by at least two distinct clones of VRE *E. faecium* in a German NICU [100]. In a US NICU, VRE-producing strains were isolated from 65 out of the 1820 admitted neonates over a three-year period. Five patients developed infections, including bloodstream infections, meningitis, and urinary tract infections. The infection-to-colonization ratio was 1:12 [101]. In contrast, in a prospective longitudinal study of a NICU in Israel, Toledano et al. detected no cases of VRE colonization among hospitalized neonates, although 61% were colonized with vancomycin-sensitive enterococci during their NICU stay. Notably, in other wards of the same hospital, 14.7% of inpatients were colonized with VRE isolates [102].

Given the emergence of VRE infections as a significant threat to NICUs, there was particular interest in developing strategies to reduce their spread and prevent outbreaks. The significance of targeted surveillance cultures was emphasized in numerous studies, which demonstrated that even a single positive culture may be associated with a substantial number of colonized infants who serve as reservoirs for VRE [103]. Indeed, as evidenced by several studies, active surveillance and cohorting, in combination with enhanced infection control measures, were highly effective in controlling NICU outbreaks [100,101,104]. Golan et al. emphasized the significant role of environmental contamination as a reservoir for VRE transmission, as evidenced by the persistence of VRE transmission facilitated by contaminated incubators despite patient cohorting and infection control measures [105].

Although *E. faecalis* is the most common strain associated with human disease, *E. faecium* was the most common strain implicated in VRE infections [96,106]. Most outbreaks in NICUs involved VanA-type VRE strains, which are associated with high-level resistance to vancomycin and teicoplanin [99,106,107]. The VanB-type is associated only with high or low-level vancomycin resistance [98]. An additional concern with the VanA phenotype is the potential for transmission of this gene and vancomycin resistance to other pathogens, such as *S. aureus* and *L. monocytogenes* [9,99]. The therapeutic management of VRE infections is particularly challenging, as vancomycin resistance often occurs with resistance to other antibiotics, including ampicillin, tetracycline, and aminoglycosides [99]. Chloramphenicol was a commonly used agent, whereas linezolid and quinupristin-dalfopristin represented newer agents that were increasingly used, despite the limited experience in neonatal use [96,99,106,107].

**Table 2:** Epidemiological, molecular and infection control measures of vancomycin-resistant enterococci in neonatal intensive care units.

Author	Country	Population	Infection/Colonization	Site of Isolation	Genotype	Clonality	Intervention
Yüce, 2001 [98]	Turkey	8	0/8	Rectal swabs	*E. faecalis* (5) *E. faecium* *E. gallinarum*	ND	ND
Rupp, 2001 [104]	US	28	0/28	Rectal, oropharyngeal swabs	*E. faecium* (VanB (25), VanA)	27/28 one clone	Cohorting, active surveillance cultures, environmental decontamination, infection control measures, reduced vancomycin use
Khan, 2002 [99]	Pakistan	4	1/3	Blood/rectal swabs	*E. faecium*, (VanA)	One clone	Cohorting, discontinuation of sharing personnel with ICU, monitoring adherence to infection control measures
Borgmann, 2004 [100]	Germany	24	1/23	Stool samples	*E. faecium* (VanA)	Two clones	Cohorting, active surveillance cultures
Singh, 2005 [101]	US	65	5/60	Blood, urine, CSF, sputum/rectal swabs	*E. faecium* (63) *E. gallinarum* (2)	Multiclonal	Cohorting, active surveillance cultures, infection control measures
Golan, 2005 [105]	UK	14	0/14	Rectal swabs	ND	One clone	ND

US: United States; UK: United Kingdom; ICU: intensive care unit; CSF: cerebrospinal fluid; ND: no data.

#### 5.3.2. 2020–2025

VRE infections remain a significant concern among hospitalized patients, and their prevalence has increased in several geographic regions compared to previous decades. In Asia, the pooled prevalence of VRE infections has been estimated at 9.1% in a systematic review, compared with 6.4% during 2000–2010 [108]. Considerable variations have been observed among European countries. However, a significant overall increase was reported in 2021 compared to 2017, from 13.4% to 17.2% [109].

Data specific to neonatal populations remain limited and mostly focus on colonization rather than infections. Recent studies continue to document VRE colonization among hospitalized neonates, with prevalence varying widely across geographic regions and institutions, from approximately 4% in the US to more than 50% in Israel in outbreak settings [110,111,112].

Invasive infection is uncommon compared with colonization, and an estimated 0–10% of colonized neonates will develop invasive infection [110,111,113]. An increased risk of necrotizing enterocolitis has been reported among colonized neonates [112,114]. Despite the common characterization of these strains as less virulent than other multidrug-resistant pathogens, the limited therapeutic options available for neonates, along with the immaturity and comorbidities of these patients, are a cause for concern in NICUs [110,113,115]. Linezolid is the most commonly used agent in VRE infections, although daptomycin is also used in selected cases [12]. The increasing use of linezolid raises concerns about the emergence of linezolid-resistant isolates. However, to date, such isolates have been reported only sporadically [115,116].

Molecular testing, including PCR-based methods and whole-genome sequencing (WGS), is increasingly used in hospital outbreaks because it provides rapid and accurate results, enabling prompt implementation of infection control measures [117]. Indeed, Saliba et al. demonstrated that PCR was associated with a median reduction of six days in turnaround time compared with rectal swab culture [118].

*E. faecium* is the most prevalent vancomycin-resistant strain of enterococci worldwide and is responsible for most outbreaks in nosocomial settings [108]. VanA type remains the most common VRE genotype. However, significant geographic variation exists, and an increased prevalence of VanB *E. faecium* has been reported in Australia and in several European countries since 2010 [119,120]. Regarding NICUs, molecular data are limited, but the existing literature reports the VanA genotype as the most frequently reported in outbreaks [111,112,113].

### 5.4. MRSA

#### 5.4.1. 2000–2005

The first documented isolate of MRSA was identified in Europe in 1962, and by the 1970s, global dissemination had occurred [121]. In 2000, over 55% of *S. aureus* strains causing nosocomial infections in intensive care units in the US were methicillin-resistant, according to the NNIS report [122]. Although MRSA infections and outbreaks in neonates had been described since the 1970s, reports of outbreaks in NICUs increased substantially during the 1990s [123]. By the 2000s, MRSA had already been recognized as a significant threat in NICUs [8,122,124,125,126,127].

The increased prevalence of MRSA led to the use of several antibiotic classes, exerting additional selective pressure and driving resistance to various non-beta-lactam antibiotic classes. Consequently, multidrug-resistant strains evolved, often retaining susceptibility to only a limited number of agents, such as vancomycin, rifampicin, mupirocin, and chloramphenicol [123,128]. Vancomycin resistance was rarely reported, yet this potential threat was already recognized [129].

The resistance of MRSA to beta-lactams is attributable to the mecA gene, which is located in the staphylococcal cassette chromosome mec (SCCmec). Seven SCCmec variants have been described, type I to VII. Until the 1990s, hospital-associated MRSA clones were implicated in nosocomial infections [121,130]. However, in the early 2000s, new, more virulent, community-associated MRSA clones emerged and were identified as causative agents in nosocomial outbreaks [121,131,132]. These clones have a different SCCmec, often carry genes encoding toxins such as Panton–Valentine leukocidin, and are susceptible to more antibiotic classes than multidrug-resistant hospital-associated MRSA clones, including several non-beta-lactam antibiotics [121,131]. The increasing number of reports of community-associated MRSA as the causative agent of outbreaks in NICUs indicated a shift in MRSA epidemiology and that these strains had become endemic in NICUs [131,132,133].

With the increasing number of MRSA outbreaks in NICUs, there was growing interest in evaluating the most effective methods for preventing transmission and eradicating MRSA. Molecular techniques, such as pulsed-field electrophoresis (PFGE), had revealed that several outbreaks were caused by a single clone, highlighting the central role of horizontal transmission in the NICU [8,125,126]. Active surveillance cultures, nasal and/or umbilical, cohorting, adherence to infection control measures, and decolonization of neonates and healthcare workers were recognized as effective measures for outbreak control [126,134,135,136]. Various approaches were used in different institutions for MRSA eradication, including intranasal mupirocin administration to colonized neonates and healthcare workers or unselectively in the NICU population, methylprosaniline chloride, hexachlorophene, or dilute povidone iodine baths with encouraging outcomes [134,135,136].

#### 5.4.2. 2020–2025

Although the prevalence of MRSA infections has decreased in several geographic regions, MRSA has become an endemic pathogen in many NICUs worldwide, associated with significant morbidity and mortality [137,138,139]. Community-associated MRSA clones, initially recognized in the early 2000s, are now well established in healthcare settings [140]. The cumulative incidence of neonatal MRSA colonization in NICUs was reported to be 7.2% in a systematic review comprising 62 studies, of which almost 94% were conducted in HICs. Significant geographic variations were detected, with the highest incidence reported in Taiwan and the lowest in Brazil [138].

Advances in molecular technologies have improved our understanding of MRSA transmission dynamics in the NICU. These technologies are increasingly used in clinical practice, enabling rapid outbreak recognition and prompt implementation of infection control measures [141]. Recent studies have documented the successful control of MRSA outbreaks in NICUs following screening with WGS and nanopore sequencing [141,142,143].

According to the CDC’s latest guidelines, active surveillance cultures for MRSA should be performed in outbreak settings. The recommended site for testing is the anterior nares. Targeted decolonization of colonized neonates alongside infection control measures should be implemented. However, no recommendation regarding the optimal decolonization agents could be made [144]. Intranasal mupirocin and chlorhexidine baths are the most common decolonization strategies in NICUs [140,145]. Nevertheless, the safety and efficacy in the neonatal population, particularly in VLBW neonates, have not been established [139,144].

Methicillin resistance may occur in isolation; however, combined resistance to other antimicrobial classes, such as fluoroquinolones, rifampicin, and clindamycin, is commonly observed [137,140]. Vancomycin is the most commonly used first-line agent for treating MRSA infections, often in combination with a beta-lactam [146]. However, the emergence of vancomycin-resistant *Staphylococcus aureus* (VRSA) and vancomycin-intermediate *Staphylococcus aureus* (VISA) is a significant concern [147]. Linezolid represents a potentially effective agent against VISA and VRSA, and resistance to linezolid is rarely observed [140].

MRSA remains an important endemic pathogen in NICUs worldwide. Advances in molecular technologies have improved outbreak detection and control, while active surveillance and decolonization remain the primary strategies for mitigating transmission. However, the emergence of multidrug-resistant strains and strains with reduced susceptibility to glycopeptides poses significant challenges in NICUs.

### 5.5. CoNS

#### 5.5.1. 2000–2005

CoNS represented the most prevalent pathogens causing LOS in the NICU, particularly affecting VLBW neonates, accounting for approximately half of all LOS episodes in the US and associated with significant morbidity [148]. As demonstrated in several studies using molecular techniques, specific CoNS clones exhibited prolonged persistence in NICUs [149,150,151]. Antimicrobial resistance was considered the primary selective force for these persistent CoNS clones, alongside virulence characteristics such as adhesion and biofilm production [150]. Multidrug resistance and biofilm formation on indwelling medical devices, such as central venous catheters, pose significant challenges to the effective treatment of invasive infections.

A high proportion of CoNS isolates resistant to commonly used antibiotics, including methicillin, were reported in NICUs [152,153]. Jain et al. reported 94% penicillin resistance and 66% methicillin resistance among 100 CoNS isolates derived from neonates with LOS in a NICU in India [153]. High proportions of methicillin-resistant CoNS isolates, up to 92%, as determined by the presence of the mecA gene, were reported in NICUs across multiple geographic regions [150,154]. Aminoglycoside resistance was reported in 19–50% of isolates [153,154]. However, beta-lactam and aminoglycoside resistance was reported to be more common in isolates implicated in LOS compared to contaminants, suggesting a possible association between antimicrobial resistance and pathogenicity [152].

A significant concern was the emergence of glycopeptide resistance among CoNS isolates [155,156]. Center et al. reported that 3.9% of CoNS isolates from colonized neonates admitted to a US NICU demonstrated decreased susceptibility to vancomycin. Prolonged hospitalization, exposure to vancomycin, and colonization with *S. warneri* strains were recognized as risk factors [156]. Although glycopeptide resistance was uncommon during this period, the widespread empirical use of vancomycin was recognized as associated with an increased risk of the emergence of glycopeptide-resistant strains.

#### 5.5.2. 2020–2025

CoNS remain the leading cause of LOS in NICUs in HICs, particularly affecting VLBW neonates [1,28]. *S. epidermidis* appears to remain the most prevalent strain associated with neonatal sepsis in many settings [157,158,159]. In addition to their ability to form biofilms, the increasing antimicrobial resistance among CoNS isolates is a major concern. Resistance to beta-lactams is widespread; methicillin resistance has been observed in up to 90% of CoNS isolates [160,161].

Glycopeptides, particularly vancomycin, are considered the first-line treatment for CoNS sepsis. However, decreased vancomycin susceptibility with increased minimum inhibitory concentration (MIC), particularly in methicillin-resistant isolates, has been observed [157,158]. A recent meta-analysis reported a 41.1% glycopeptide heteroresistance among CoNS isolates, raising concerns about the efficacy of glycopeptides as first-line agents [162].

### 5.6. A. baumannii

#### 5.6.1. 2000–2005

*A. baumannii*, a Gram-negative coccobacillus, was increasingly recognized as an important nosocomial pathogen in the early 2000s, particularly in intensive care units [163]. Outbreaks in NICUs were sporadically reported [164,165,166]. Although resistance to carbapenems, aminoglycosides, and fluoroquinolones was rarely reported, concerns had already been raised about the emergence of multidrug-resistant strains due to the selective pressure exerted by the widespread use of these broad-spectrum antibiotics [163,166].

#### 5.6.2. 2020–2025

Multidrug-resistant *A. baumannii* has emerged as an important cause of nosocomial infections and outbreaks in NICUs, particularly in LMICs [7,167,168]. *A. baumannii* has the capacity to survive in the hospital environment for a prolonged time and the potential for clonal spread [168,169]. Moreover, it has acquired resistance to various antibiotic classes, including cephalosporins, aminoglycosides, fluoroquinolones, and carbapenems [168]. Of particular concern is the increasing prevalence of carbapenem-resistant isolates, primarily mediated by oxacillinase genes, particularly OXA-23 [167,170]. The treatment options for multidrug-resistant *A. baumannii* are limited, and colistin has been widely used. However, the emergence of colistin-resistant *A. baumannii* strains is an increasing concern [171,172].

*A. baumannii* is estimated to be implicated in 1–6% of neonatal sepsis cases; however, uneven geographical distribution is observed, with a notably higher incidence in African and Asian countries [167]. According to a large observational study across 11 countries, primarily in Africa and Asia, *Acinetobacter* was identified as the third most prevalent causative agent of neonatal sepsis, accounting for 12.8% of cases. More than 70% of the isolates were resistant to meropenem [38]. In a NICU in South Africa, 13% of culture-confirmed sepsis cases were attributed to *A. baumannii*. In this study, 17% of the isolates were identified as extremely drug-resistant, displaying susceptibility exclusively to colistin [168]. In Ethiopia, all *A. baumannii* isolates associated with neonatal sepsis demonstrated multidrug resistance [173].

## 6. Evolution of Diagnostic Technologies in Neonatal Sepsis

Blood and/or other sterile-fluid cultures and conventional susceptibility testing were the main diagnostic tools in the early 2000s. Although culture from a sterile body site remains the gold standard for sepsis diagnosis, it has significant limitations. These include low sensitivity, which depends on the blood volume obtained, and a long turnaround time of 24–72 h [174,175]. The delay in detecting pathogens may lead to unnecessary broad-spectrum antibiotic administration and prolonged courses, which can contribute to antimicrobial resistance [1].

Although molecular techniques were already employed in the early 2000s, their use was limited, primarily for research purposes, such as identifying specific resistance genes and investigating outbreaks [44,100,107,124,130,176]. Molecular epidemiological studies primarily relied on PFGE, which was regarded as the most accurate typing technique for assessing the genetic relatedness of isolates and determining clonal transmission during outbreaks [104,121,176].

Over the past two decades, significant advances have been made in molecular diagnostics. Novel techniques, including multiplex PCR assays, T2 Magnetic Resonance (T2MR) technology, matrix-aided laser desorption/ionization time-of-flight mass spectrometry (MALDI-TOF MS), and WGS have significantly improved pathogen identification and characterization and outbreak surveillance [1,141,142,143,174,175]. Utilization of these techniques can guide clinical decisions and limit the prolonged empirical antibiotic use. However, the increased cost and the limited availability of these techniques significantly reduce their widespread use [1]. Optimization of molecular methods, wider availability across institutions, and the development of new methods to assess pathogen virulence and antimicrobial susceptibility are future goals to improve sepsis diagnosis and outbreak control [174].

## 7. Evolution of Antimicrobial Therapy

Over the past two decades, empirical antibiotic treatment regimens for EOS and LOS in NICUs have remained largely unchanged. However, the increasing antimicrobial resistance has driven important changes, including the introduction of newer agents and the implementation of antimicrobial stewardship programs.

Despite the introduction of novel antimicrobial agents against multidrug-resistant pathogens, treating multidrug-resistant infections in NICUs remains challenging due to the limited therapeutic options approved for neonates. Novel beta-lactam/beta-lactamase inhibitor combinations, including ceftazidime/avibactam and ceftolozane/tazobactam, have been recently approved for use in neonates and are effective against multidrug-resistant Gram-negative pathogens [70]. Older drugs, such as colistin and fosfomycin, have also been reintroduced into clinical practice for the treatment of multidrug-resistant pathogens [172].

Although the association between broad-spectrum antibiotic use, prolonged antibiotic courses, and the emergence of antimicrobial resistance had already been recognized in the early 2000s, the systematic implementation of antimicrobial stewardship programs in NICUs has largely occurred in recent years [177]. These programs promote a more individualized approach to antibiotic therapy, including early discontinuation of antibiotics in cases of unconfirmed sepsis, de-escalation of therapy based on susceptibility results, minimization of treatment duration, and empirical antibiotic coverage informed by local resistance patterns [6,13].

Evidence from studies on the implementation of antimicrobial stewardship programs in the NICU is encouraging. Kahn et al. reported a 34.1% reduction in antibiotic use at NICU admission and a 45.3% reduction in antibiotic use beyond 72 h following the implementation of a stewardship program [178]. Several studies have demonstrated that a reduction in the duration of antibiotic treatment can be achieved through stewardship measures [178,179,180,181,182]. A recent meta-analysis of 70 studies reported a significant reduction in antibiotic initiation, duration of therapy, and antibiotic administration for more than five days. Overall, a 20% reduction in antibiotic days and a two-day reduction in treatment duration were observed [177].

## 8. Discussion

Antimicrobial resistance is not a recently emergent phenomenon; rather, it is a persistent and increasing threat. As early as 1945, Alexander Fleming predicted that the inappropriate use of penicillin could lead to the emergence of resistant species. His prediction was confirmed in the early 1950s, when penicillin-resistant *S. aureus* had emerged and disseminated globally [183].

The comparison between the two periods, separated by two decades, highlights that antimicrobial resistance is an evolutionary phenomenon, driven by the selective pressure exerted by antimicrobial prescribing patterns (Table 3). Across the studies included in this review, the evolution of resistance is evidenced by the emergence of ESBLs, which can be associated with the widespread use of ampicillin and third-generation cephalosporins, and by the subsequent emergence of carbapenem-resistant strains in settings with increased carbapenem use. Similarly, the extensive use of vancomycin to treat MRSA has led to the recent emergence of VISA and VRSA strains. This represents the most important lesson from this comparison: the extensive use of an effective drug or even the development of a new agent does not prevent the emergence of resistance. Instead, preserving the effectiveness of antibiotics through rational use remains the most effective strategy to limit the emergence of resistant pathogens [33,177]. The consequences of irrational antibiotic use are becoming increasingly apparent. Several pathogens regarded as emerging two decades ago are now considered endemic in many NICUs, particularly in regions with a high burden of antimicrobial resistance, such as LMICs, and multidrug-resistant pathogens have become increasingly common.

In the last decade, considerable efforts have been made in many NICUs worldwide to implement antimicrobial stewardship programs and limit unnecessary antibiotic use. A growing number of institutions are organizing coordinated interventions through multidisciplinary teams of neonatologists, infection control specialists, microbiologists and pharmacists. These interventions aim to monitor antibiotic prescribing patterns, conduct surveillance of antimicrobial resistance and perform periodic audits for feedback on antibiotic use. Recently, a number of national and international surveillance networks have been developed, including the NeoIPC program, a consortium that includes fourteen institutions from eleven European and African centers. The primary objectives of this consortium are to perform infection surveillance, monitor resistance trends, and provide standardized methods and reference data to support infection prevention and stewardship programs [184,185].

However, the effective implementation of antimicrobial stewardship in NICUs is primarily challenged by the nonspecific clinical presentation of neonatal sepsis and the lack of specific biomarkers in daily clinical practice [186,187]. Because prompt administration of antibiotic therapy is critical for survival and reducing morbidity, empirical antibiotic therapy is routinely administered in suspected cases of sepsis, and prolonged antibiotic courses are also frequently given despite negative cultures [183,186,188,189]. There is growing interest in molecular methods that could enhance the diagnosis of neonatal sepsis, enable earlier identification of pathogens and their resistance patterns than with available biomarkers, and facilitate targeted antibiotic use. Novel molecular methods, including PCR-based techniques and WGS, have proven to be promising tools for improved diagnostic precision, outbreak investigation, and surveillance of the evolution of resistance. Whilst these techniques are currently utilized as adjuncts to conventional methods to improve diagnostic accuracy, further optimization and widespread availability are imperative before they can replace conventional methods such as cultures [175]. Τhe ideal biomarker with high sensitivity and specificity has not yet been identified and should be prioritized in future studies, as it is a prerequisite for targeted antibiotic use [29].

A further challenge to antimicrobial stewardship in neonates is the limited number of antibiotics approved for neonatal use against multidrug-resistant pathogens. Although several novel agents have been approved against multidrug-resistant sepsis for adult patients, with few exceptions, these agents are not labeled for use in neonates [183]. Cefiderocol, meropenem/vaborbactam, and imipenem/relebactam have been shown to be effective agents for treating multidrug-resistant sepsis in adults; however, data in neonates are sparse and limited to case reports or case series [12,146,190]. Two novel beta-lactam/beta-lactamase inhibitor combinations, ceftazidime/avibactam and ceftolozane/tazobactam, which are highly effective against multidrug-resistant Gram-negative pathogens, have recently been approved for use in neonatal populations. Despite the progress made over the past two decades and the expansion of therapeutic options for neonates, there remains a pressing need for further research and development. It is urgent that clinical trials be conducted in neonates to provide a basis for the safe and effective use of these agents. This will provide clinicians with safer and more effective options for resistant infections.

The burden of antimicrobial resistance is not uniformly distributed worldwide. The burden of multidrug-resistant Gram-negative neonatal sepsis is disproportionately higher in LMICs compared to HICs [7]. Although ESBLs and CRE represent global challenges in NICUs, their prevalence is considerably higher in LMICs [11]. The regional differences are probably multifactorial, reflecting variations in infection prevention strategies, access to diagnostic methods, availability of antibiotics and effective implementation of antimicrobial stewardship. Thus, regional surveillance data should be considered and treatment should be based on local resistance patterns.

The available evidence included in this review is mostly derived from observational studies, outbreak-investigation series, and single-center cohorts, which show significant heterogeneity not only in study design, but also in infection control practices, antibiotic prescribing patterns and the availability of diagnostic methods between different institutions. This heterogeneity makes direct comparison between studies difficult and should be considered when interpreting resistance rates and pathogen distributions. In addition, the observed epidemiological changes over time should be interpreted with consideration of advances in molecular technologies used in recent epidemiological studies, which are expected to enhance pathogen detection and outbreak recognition. Therefore, although the evolution of antimicrobial resistance that is observed between the two study periods is likely largely driven by selective pressure resulting from antibiotic use, changes in infection prevention practices and the availability of advanced techniques should also be considered.

Clinicians play a central role in limiting the emergence and spread of antimicrobial resistant pathogens. Limiting unnecessary antibiotic exposure, de-escalation of therapy, minimizing treatment duration, and using empirical antibiotic regimens based on local resistance patterns are the cornerstones of antimicrobial stewardship programs and should be implemented in routine clinical practice. Of particular importance is strict adherence to infection prevention and control measures by clinicians, complemented by continuous surveillance and rapid outbreak recognition and control. These represent essential components in limiting horizontal transmission of multidrug-resistant pathogens in the NICU. Collaborative relationships between NICUs at national and international levels, together with the expansion of neonatal surveillance networks, are necessary to monitor epidemiological data and emerging resistance trends and to offer tailored empirical treatment protocols and infection prevention strategies.

Infection prevention and the rational use of antibiotics are key to disrupting the vicious cycle of resistance. As early as 2000, the CDC recognized that preventing antimicrobial resistance comprises four components: infection prevention, effective diagnosis and treatment, appropriate antibiotic use, and prevention of transmission [183]. Although these principles have been recognized for decades, antimicrobial resistance has continued to emerge and disseminate, highlighting implementation gaps. It is therefore imperative to strengthen adherence to infection prevention and antimicrobial stewardship to effectively limit the future burden of resistance.

## 9. Limitations

This review has several limitations. Although an extensive literature search was conducted during the preparation of this review, given its narrative nature, a standardized literature search was not performed, which could potentially lead to selection bias. Another limitation of this narrative review is that it compares two selected five-year periods, rather than providing a continuous assessment of literature throughout the entire interval. Consequently, the findings should be interpreted as a comparison between two representative periods rather than as temporal trends across two decades. Moreover, the included studies show significant heterogeneity regarding geographical region, study design, population, and outcomes, limiting direct comparison between studies.

## 10. Conclusions

Over the past few decades, antimicrobial resistance has significantly challenged the management of neonatal sepsis. Broad-spectrum antibiotic use and prolonged antibiotic courses are established factors predisposing to the emergence of multidrug-resistant pathogens.

Antimicrobial resistance is not static but rather an evolutionary process, driven by the selective pressure exerted by widely used antibiotics in a particular era. In the early 2000s, although resistance to aminoglycosides was uncommon, concerns were raised about the efficacy of standard empirical regimens for neonatal sepsis. The emergence of resistant pathogens in NICUs, including ESBL-producing *Enterobacterales*, VRE, MRSA, and CoNS, was associated with increasingly reported outbreaks in institutions worldwide. The significance of horizontal pathogen transmission in the outbreak context was established, and prevention measures were implemented in clinical practice, including active surveillance cultures, cohorting and infection control measures.

The increasing prevalence of antimicrobial resistance was associated with widespread use of vancomycin, third and fourth-generation cephalosporins, and carbapenems, further driving the selection and emergence of resistant strains. In the 2020s, the widespread dissemination of multidrug-resistant pathogens has been documented across many regions worldwide, with the highest burden reported in NICUs in LMICs. MRSA and ESBL-producing *Enterobacterales* have become endemic in many NICUs. The increased use of carbapenems in NICUs has contributed to the emergence of carbapenem-resistant pathogens. The widespread use of vancomycin for the treatment of MRSA and CoNS has contributed to the increasing prevalence of VRE and the emergence of VRSA and VISA.

Despite novel antimicrobial agents and the repurposing of older drugs, the management of multidrug-resistant sepsis in neonates remains challenging, as the therapeutic options are limited and multidrug-resistant sepsis is associated with significant morbidity and mortality. Reducing inappropriate and unnecessary antibiotic use is considered the most effective strategy for mitigating antimicrobial resistance. In recent years, antimicrobial stewardship programs, together with advances in molecular diagnostic techniques that enable rapid pathogen identification, have become key strategies for reducing the selective pressure exerted by antibiotics and improving outbreak control in NICUs.

The comparison between these two representative periods provides several important insights that can inform future priorities for clinical practice and research. One of the most important observations is that the sequential emergence of ESBL-producing pathogens, CRE, and VISA/VRSA suggests that changes in antibiotic prescribing patterns contribute to the evolution of antimicrobial resistance. Thus, rational and targeted antibiotic use, together with continuous evaluation of empirical antibiotic regimens based on local surveillance data, is essential to preserve the effectiveness of existing antibiotics. National and international surveillance networks should be strengthened and expanded to ensure coordinated responses to emerging resistance threats. Another important finding is that pathogens considered emerging in the 2000s are now endemic in many NICUs, highlighting the importance of active surveillance and strengthening of infection prevention strategies. Advances in molecular diagnostic techniques have substantially improved pathogen identification, susceptibility testing and outbreak investigation. Further optimization and wider availability of these techniques should remain a priority. Although the development of new antibiotics effective against multidrug-resistant pathogens is necessary, this alone cannot overcome antimicrobial resistance. Future research should therefore prioritize clinical and pharmacokinetic trials in neonatal populations to develop new agents effective against multidrug-resistant pathogens, together with the identification of reliable biomarkers to enhance timely diagnosis and targeted antibiotic therapy. Preserving the effectiveness of existing antibiotics through antimicrobial stewardship remains equally important.

A coordinated strategy that combines enhanced surveillance, infection prevention, antimicrobial stewardship, and research focused on the diagnosis and management of multidrug-resistant neonatal sepsis is warranted to limit the spread of multidrug-resistant pathogens and the emergence of new resistant strains.

## Figures and Tables

**Table 3:** Comparison of epidemiology, resistance patterns and therapeutic options of main multidrug-resistant pathogens in neonatal intensive care units between 2000–2005 and 2020–2025.

Pathogen	2000–2005			2020–2025		
	Epidemiology	Resistance Mechanisms/Patterns	Therapeutic Options	Epidemiology	Resistance Mechanisms/Patterns	Therapeutic Options
ESBL	Emerging, increasingly reported in NICU outbreaks	TEM-, SHV, emerging CTX-M genotype	Carbapenems	Increased prevalence, often endemic in LMICs	CTX-M predominant	Carbapenems Ceftazidime/avibactam Ceftolozane/tazobactam
CRE	Sporadic reports	Often non-carbapenemase-mediated (ESBL/AmpC + porin loss)	Colistin	Increasing global dissemination, particularly in LMICs	Widespread carbapenemase-mediated resistance (NDM, OXA-48, KPC, VIM)	Colistin Ceftazidime/avibactam
*A. baumannii*	Sporadic outbreaks	Low resistance rates	Carbapenems	Increased prevalence, particularly in LMIICs	MDR/XDR strains, OXA-type carbapenemases	Colistin
VRE	Emerging outbreaks in NICUs	*E. faecium*, VanA type predominant	Chloramphenicol Linezolid (emerging) quinupristin-dalfopristin (off-label)	Persistent nosocomial pathogen	*E. faecium*, VanA type predominant, increased VanB	Linezolid Daptomycin (off-label)
MRSA	Outbreaks in NICUs globally	HA clones predominated, CA clones emerged in outbreaks	Vancomycin Linezolid (emerging) Chlorampenicol	Endemic in many NICUs	Mixed and HA clones, emergence of VRSA and VISA strains	Vancomycin Linezolid Daptomycin (off-label)
CoNS	Leading cause of LOS	High methicillin resistance (mecA)	Vancomycin	Leading cause of LOS	Methicillin resistance (mecA), increasing glycopeptide heteroresistance	Vancomycin Linezolid

ESBL: expended spectrum beta-lactamases; CRE: carbapenem-resistant *Enterobacterales*; VRE: Vancomycin-resistant *Enterococcus*; MRSA: methicillin-resistant *Staphylococcus aureus*; CoNS: Coagulase-negative *Staphylococcus*; LMICs: Low- and middle-income countries; KPC: *Klebsiella pneumoniae* carbapenemase; NDM: New Delhi metallo-beta-lactamase; OXA: oxacillinase; VIM: Verona integron-encoded metallo-beta-lactamase; HA: hospital-acquired; CA: community-acquired; VRSA: vancomycin-resistant *Staphylococcus aureus*; VISA: vancomycin-intermediate *Staphylococcus aureus*; LOS: late-onset sepsis.

## Data Availability

No new data were created or analyzed in this study. Data sharing is not applicable to this article.
